# Green synthesis of silver nanoparticle prepared with *Ocimum* species and assessment of anticancer potential

**DOI:** 10.1038/s41598-024-61946-y

**Published:** 2024-05-22

**Authors:** Asha Monica Alex, Senthilkumar Subburaman, Shikha Chauhan, Vishal Ahuja, Gholamreza Abdi, Maryam Abbasi Tarighat

**Affiliations:** 1 Department of Biotechnology, St Joseph’s College, (Autonomous) affiliated to Bharathidasan University, Trichy, Tamil Nadu India; 2 Department of Botany, St Joseph’s College, (Autonomous), Trichy, Tamil Nadu India; 3 University Institute of Biotechnology, Chandigarh University Mohali (Punjab), Gharuan, India; 4 University Institute of Biotechnology and University Centre for Research and Development Chandigarh University Mohali (Punjab), Gharuan, India; 5 Department of Biotechnology, Persian Gulf Research Institute, Persian Gulf University, Bushehr, 75169 Iran; 6 Faculty of Nano and Bio Science and Technology, Persian Gulf University, Bushehr, 75169 Iran

**Keywords:** Green synthesis, Silver nanoparticles, Anticancer activity, Antimicrobial activity, MCF-7 cells, Biochemistry, Cancer, Plant sciences

## Abstract

Silver nanoparticles (AgNPs) have gained much attention due to their unique physical, and chemical properties. Integration of phytochemicals in nanoformulation might have higher applicability in healthcare. Current work demonstrates the synthesis of green AgNPs with *O. gratissimum* (gr-AgNPs) *O. tenuiflorum* (te-AgNPs) and *O. americanum* (am-AgNPs) followed by an evaluation of their antimicrobial and anticancer properties. SEM analysis revealed spherical-shaped particles with average particle sizes of 69.0 ± 5 nm for te-AgNPs, 46.9 ± 9 nm for gr-AgNPs, and 58.5 ± 18.7 nm for am-AgNPs with a polydispersity index below 0.4. The synthesized am-AgNPs effectively inhibited *Klebsiella pneumonia, Escherichia coli, Staphylococcus aureus, Aspergillus niger,* and *Candida albicans* with 23 ± 1.58 mm, 20 ± 1.68 mm, 22 ± 1.80 mm, 26 ± 1.85 mm, and 22 ± 1.40 nm of zone of inhibition respectively*.* Synthesized AgNPs also induced apoptotic cell death in MCF-7 in concentration-dependent manner. IC_50_ values for am-AgNPs, te-AgNPs, and gr-AgNPs were 14.78 ± 0.89 µg, 18.04 ± 0.63 and 15.41 ± 0.37 µg respectively which suggested that am-AgNPs were the most effective against cancer. At higher dose size (20 µg) AgNPs were equally effective to commercial standard Doxorubicin (DOX). In comparison to te-AgNPs and gr-AgNPs, am-AgNPs have higher in vitro anticancer and antimicrobial effects. The work reported *Ocimum americanum* for its anticancer properties with chemical profile (GCMS) and compared it with earlier reported species. The activity against microbial pathogens and selected cancer cells clearly depicted that these species have distinct variations in activity. The results have also emphasized on higher potential of biogenic silver nanoparticles in healthcare but before formulation of commercial products, detailed analysis is required with human and animal models.

## Introduction

Antibiotics and antimicrobial drugs have become inseparable parts of healthcare used for managing infectious disorders and preventing infection during difficult surgeries. However, overexploitation of antibiotics resulted in the evolution of drug resistance strains of pathogens^1^ that directly affect hospitalization phase and mortality rates^2,3^. Similar observations have been recorded in the cases of cancer chemotherapy as drug resistance cases have increased due to its frequent application. It also results in the unchecked development of transformed cells and dynamic genomic alterations that give rise to malignant traits in normal cells^4^. Besides drug resistance, cancer therapy also faces issues with delayed diagnosis, non-specific systemic distribution, insufficient or high drug dosages, poor drug delivery, and difficulty in tracking therapeutic outcomes^5^.

The present cancer therapy challenges can be addressed through nanotechnology and nanoformulations^6,7^. Nanomaterials have shown peculiar natures and characteristics such as optical, magnetic, chemical, and mechanical properties, that allow multifaceted applications in food, healthcare, and agriculture^8,9^. Metal-based particles like gold (AuNPs) and silver (AgNPs) have drawn considerable interest in infectious diseases and post-surgical infections due to inherent bioactivities and lower possibility of toxicity to normal cells^10,11^. Due to their unique stability, varied geometries, and simplicity of AgNPs to obstruct bacterial cell growth, AgNPs have extensive usage in the construction of multi-resistant drugs^12^, speed up the healing process^13^, ointment balms^14^, anticancer agents^15^, and dental fillers^16^.

Conventional NPs synthesis methods involve the utilization of toxic and dangerous materials and pose a high risk of poisoning^17^. Green NPs synthesis by using plant extract and natural reducing materials has offered a cost-effective, eco-friendly approach with a high level of reproducibility^18,19^. Tulsi (holy basil), one of the most imperative herbal plants from the Lamiaceae family is widely used as food and a dominant ingredient of healthcare formulations^20^. It is native to the Indian subcontinent and has extensively been utilized in Ayurveda and is frequently referred to as an "Elixir of Life"^21^. The medicinal properties of tulsi led to its use in treating epilepsy^22,23^, asthma^24^, cough^25^, anemia^26^, parasite infections^27,28^, neuralgia^29^, and inflammation^30^. Tulsi leaf extracts are recommended in the Indian Materia Medica for the treatment of pyrexia, rheumatism, and bronchitis^31^ while the tea infusion has been used to treat hepatic and gastrointestinal diseases, the leaf juice has been utilized topically to cure earaches, malaria, snakebites, and mosquito bites^32^. In the current work, AgNPs were synthesized with aqueous leaf extracts of *O. gratissimum*, *O. tenuiflorum,* and *O. americanum* and compared for antimicrobial and in vitro anticancer activities.

## Results

**Figure 1:**
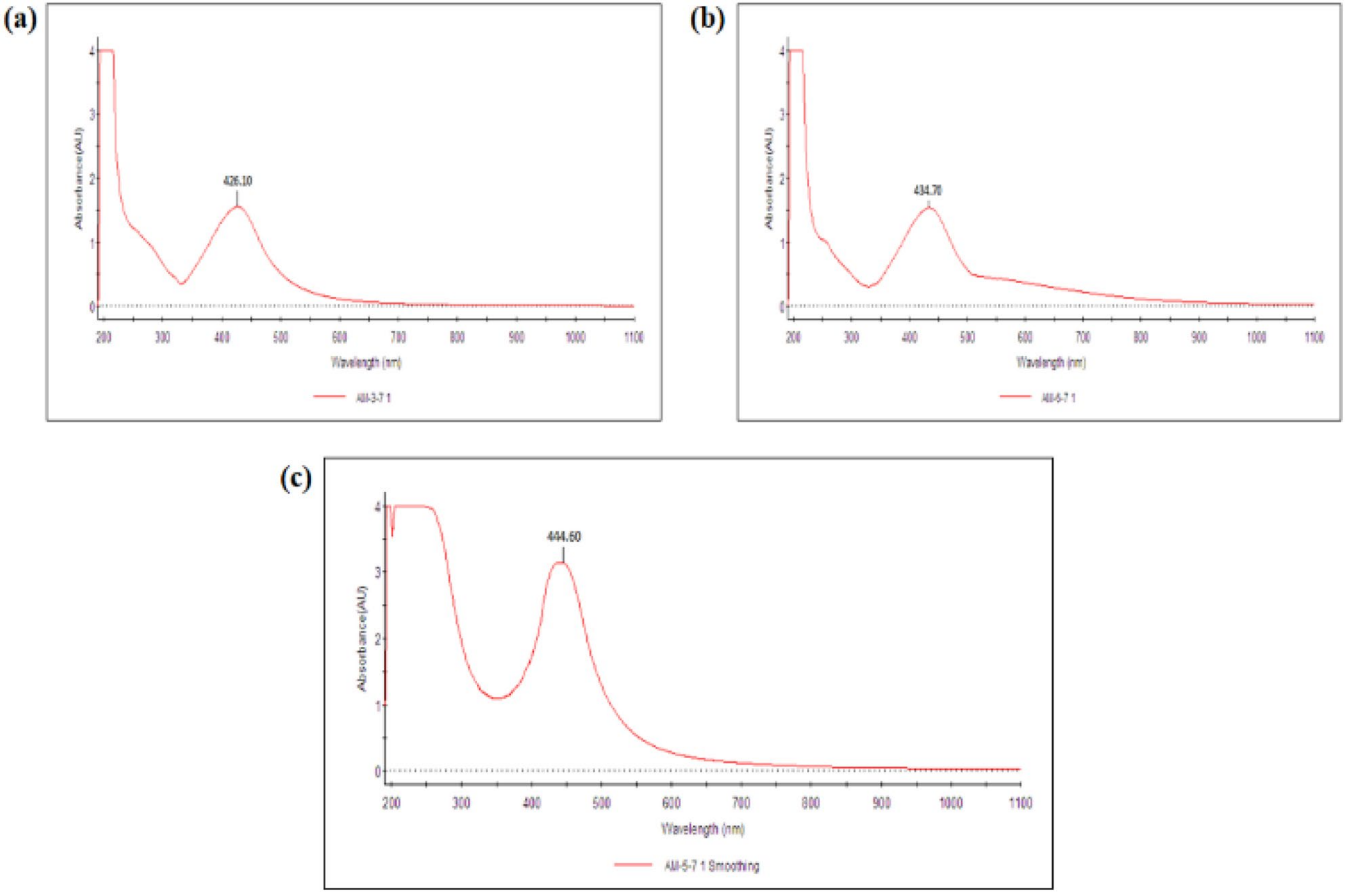
UV–visible spectroscopic analysis of synthesized AgNPs from the *O. tenuiflorum*, *O. gratissimum*, and *O. americanum* leaf extracts.

The biogenic nanoparticles i.e. te-AgNPs, am-AgNPs, and gr-AgNPs were prepared from AgNO_3_ as a precursor using extract of different species of *Ocimum*. The basic concept behind AgNPs synthesis is the use of plant extract as a reducing and stabilizing agent^33,34^. During the nanoparticle synthesis process, initially, the emergence of a dark brown hue in aqueous solutions of (te), (gr), and (am) signifies the formation of te-AgNPs, am-AgNPs, and gr-AgNPs, respectively^35^. During the interaction, some of the biomolecules from plant extract involved in the reduction of Ag^+^ ions into Ag^0^ reduced or metallic form of silver followed by their stabilization by biomolecules present in the same extract to yield silver nanoparticles. Bioactive compounds present in the extracts of *Ocimum* species such as alkaloids, saponins, flavonoids, phenols, tannins, and terpenoids are actively involved in the reduction and stabilization of nanoparticle synthesis^35^. Previous literature also supported the fact that phytochemicals can act as reducing agents as well as capping agents. Phenolics and flavonoids are known to act as reducing agents while xanthones and phloroglucinols act as capping agents in nanoparticle synthesis^36^. The confirmation of silver nanoparticle synthesis was done through UV–visible spectroscopy. The surface plasmon resonance (SPR) peaks of te-AgNPs, gr-AgNPs, and gr-AgNPs were observed at 426.10 nm (Fig. 1a), 434.70 nm (Fig. 1b), and 444.60 nm (Fig. 1c), respectively. The SPR peaks for each of the silver nanoparticles were in accordance with the previous literature. The correlation of SPR band of synthesized silver nanoparticles has been established in section "UV–visible spectroscopic analysis".

### Characterization of synthesized AgNPs

#### UV–visible spectroscopic analysis

UV–visible spectrophotometer serves as an important tool to determine if the plant extract can reduce silver salt to NPs. Following the blending of the extract and silver nitrate solution, UV absorption spectra were measured.

The UV–visible spectrum analysis of synthesized AgNPs demonstrated the maximum absorbance peak at 426.10 nm for te-AgNPs from *O. tenuiflorum* (Fig. 1a), 434.70 nm for gr-AgNPs from *O. gratissimum* (Fig. 1b), and 444.60 nm for am-AgNPs from *O. americanum* (Fig. 1c), respectively, indicating the occurrence of surface plasmon resonance (SPR). The similar peak shifting and occurrence of SPR was also reported in earlier literature with silver nanoparticles^37,38^. Li et al. (2023) showed that AgNPs exhibited local SPR due to scattering of radiated light on particle surface^38^. In another work, Benjamin et al. (2006) proved that SPR in AgNPs and peak position depend upon the shape of the particle. Sharp peaks around 400–450 nm can be observed in the case of spherical nanoparticles while cubical, octahedron, and tetrahedron-shaped particles have multiple peaks of different intensities due to multiple dipole symmetries^39^.

#### SEM analysis

**Figure 2:**
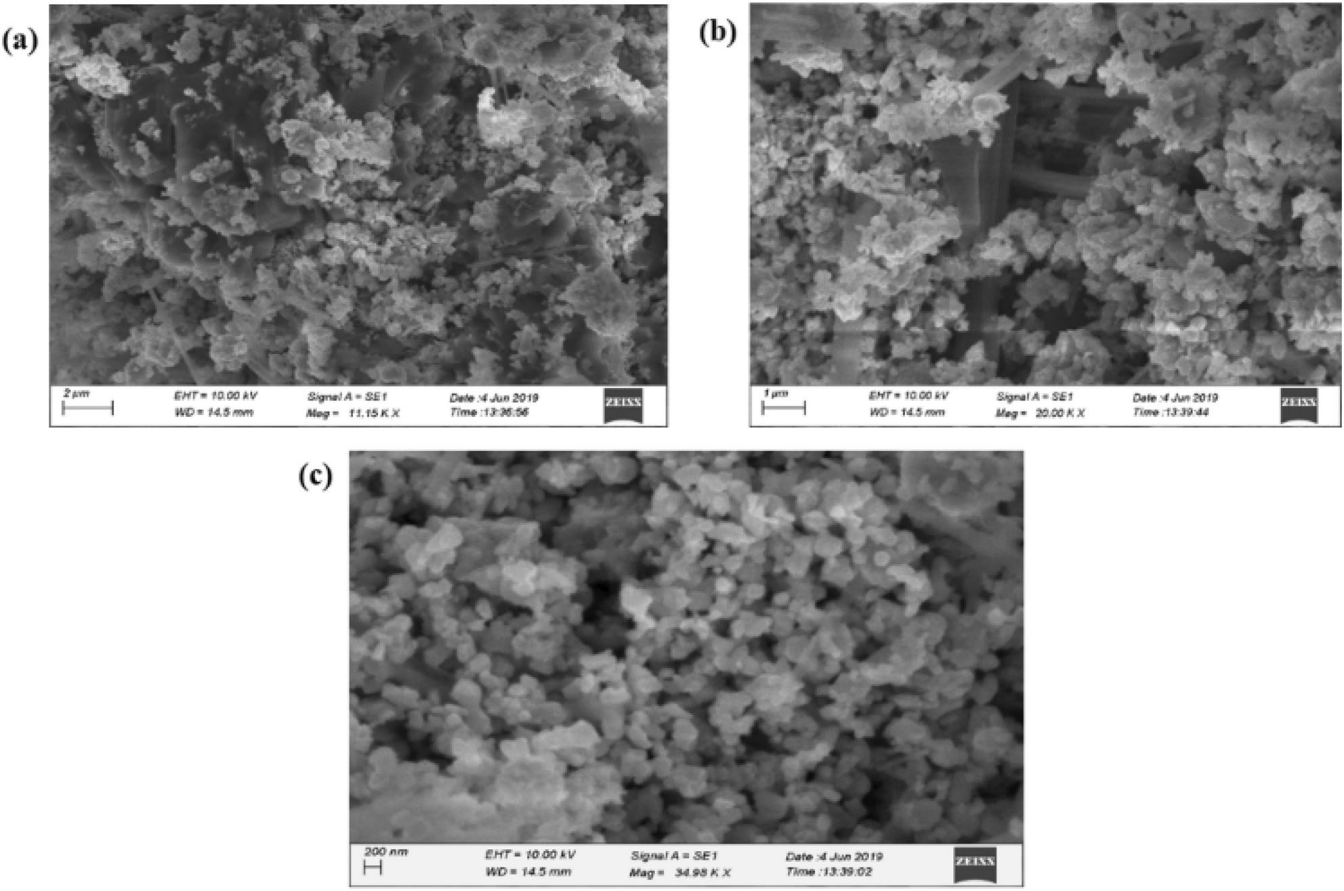
SEM analysis of synthesized AgNPs from the *O. tenuiflorum* (**a**), *O. gratissimum* (**b**), and *O. americanum* (**c**) leaf extracts.

SEM analysis proved that the synthesized AgNPs are rocky surfaced spherical morphology was found in te-AgNPs from *O. tenuiflorum* (Fig. 2a) and flake surfaced spherical shape was found in gr-AgNPs from *O. gratissimum* (Fig. 2b) whereas spherical shape was observed in am-AgNPs from *O. americanum* (Fig. 2C). The morphology and molecule size of formulated AgNPs were studied by utilizing SEM investigation. The SEM micrographs of AgNPs recommended the amalgamation of mono-scattered AgNPs of high thickness. The micrographs additionally uncovered consistency in the size and shape of the orchestrated AgNPs.

#### DLS analysis

**Figure 3:**
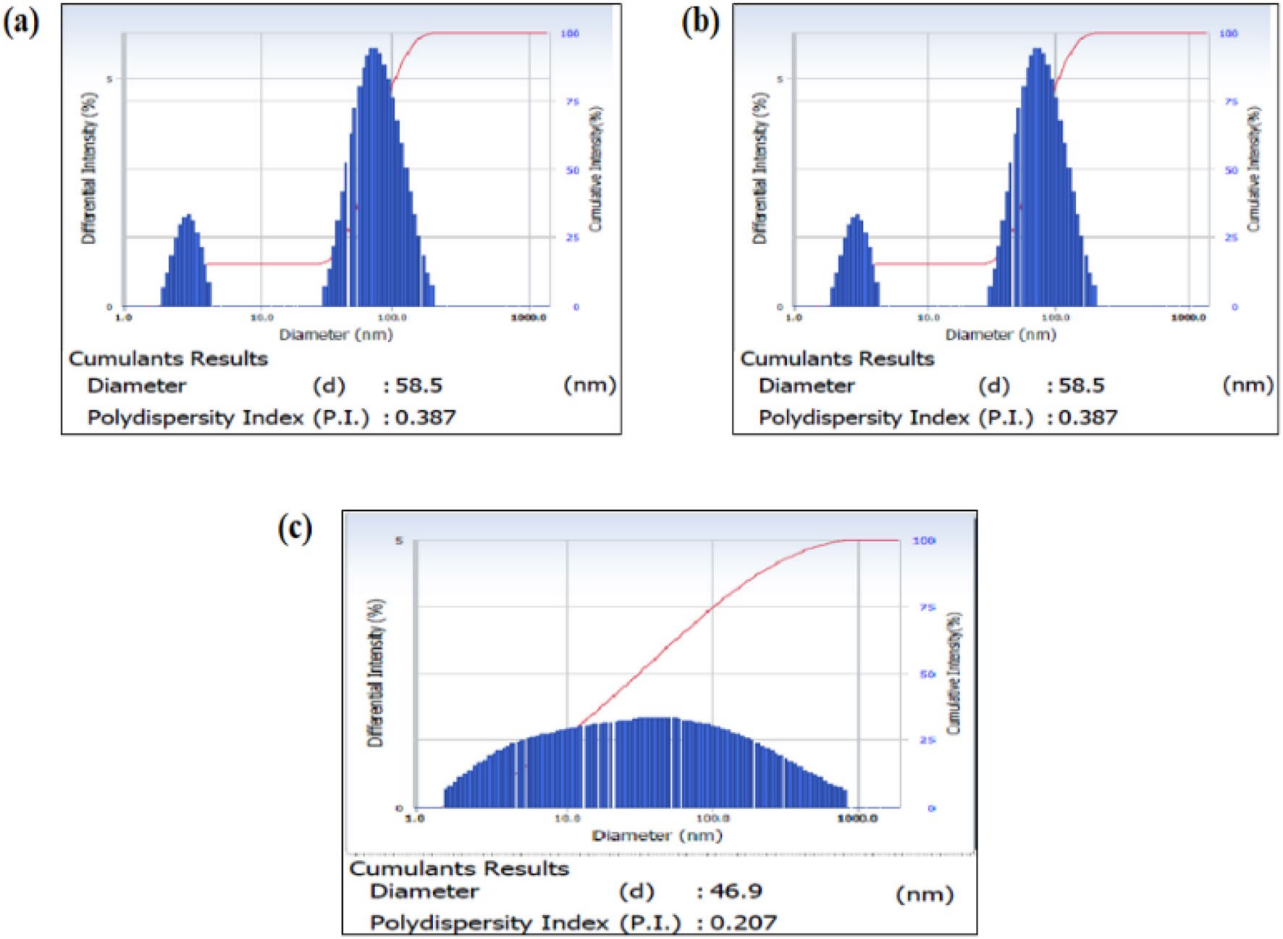
DLS analysis of synthesized AgNPs from the *O. tenuiflorum* (**a**), *O. gratissimum* (**b**), and *O. americanum* (**c**) leaf extracts.

The DLS study revealed the size distribution of fabricated te-AgNPs, gr-AgNPs, and am-AgNPs. The average diameter of te-AgNPs was 69.0 ± 5.4 nm (Fig. 3a), 46.9 ± 9.0 nm for gr-AgNPs (Fig. 3b), and 58.5 ± 18.7 nm from am-AgNPs (Fig. 3c). The findings of the DLS study of te-AgNPs showed the zeta average diameter of 69.0 nm with PI value of 0.356. Whereas gr-AgNPs have a zeta average diameter of 46.9 nm with a PI value of 0.207 and am-AgNPs have a zeta average diameter of 58.5 nm with a PI value of 0.3870 were observed.

#### FT-IR analysis of AgNPs from *Ocimum* species

**Figure 4:**
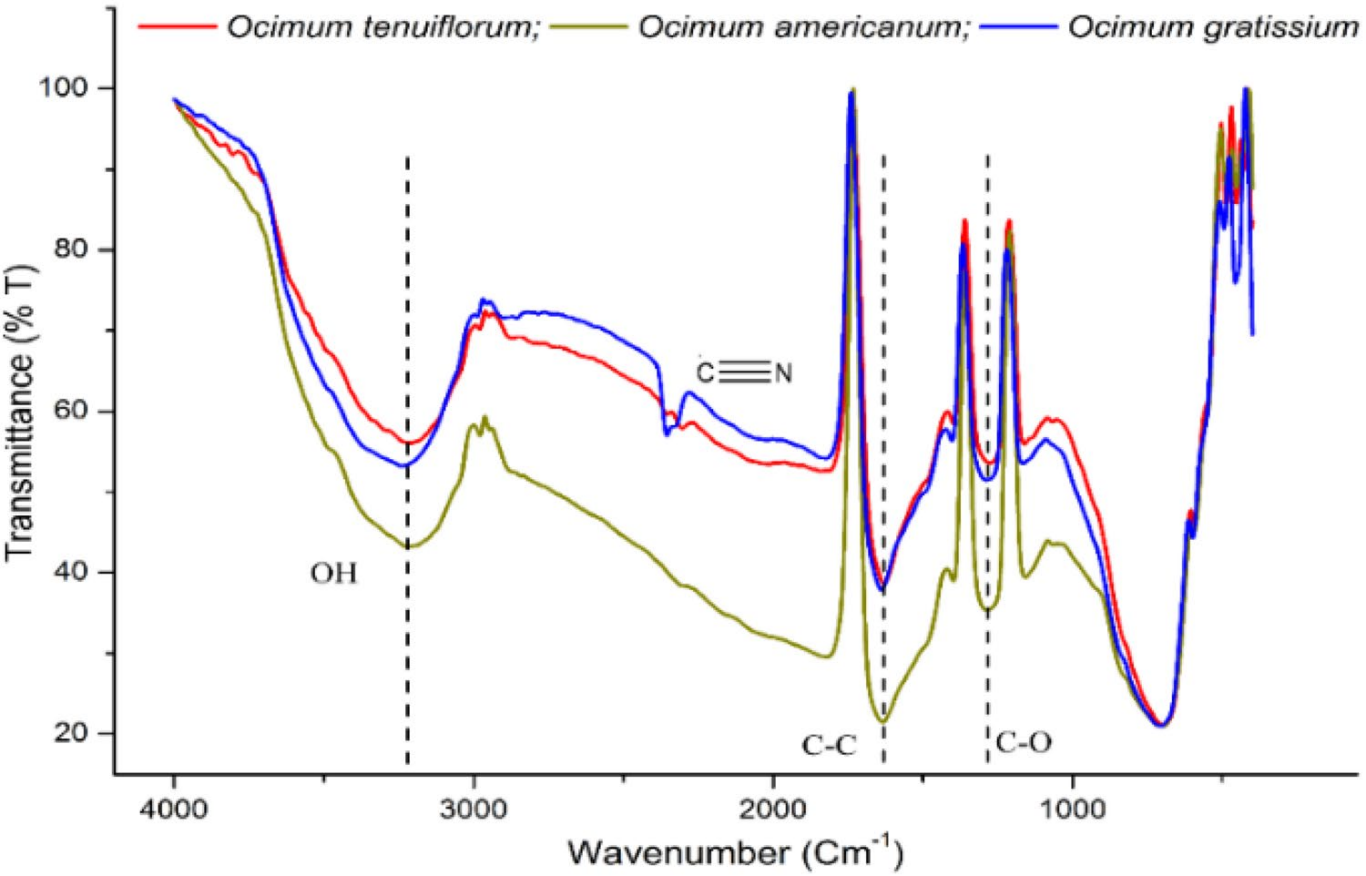
FT-IR analysis of synthesized AgNPs from the *O. tenuiflorum* (**a**), *O. gratissimum* (**b**), and *O. americanum* (**c**) leaf extracts.

FT-IR assessments were performed to detect the functional groups that present on the formulated te-AgNPs, gr-AgNPs, and am-AgNPs. The peaks obtained for fabricated te-AgNPs, gr-AgNPs, and am-AgNPs from different *Ocimum* species were revealed in Fig. 4. Almost the same with slight variations at absorbed wavelengths and percentage transmittance to elucidate the functional groups and the metabolites that have reduced silver ions. It was apparent that strong peaks obtained at 3311 cm^-1^ and 3342 cm^-1^ resemble O–H stretching. The peak at 2947 cm^-1^ and 2077 cm^-1^ resembles carboxylic acid O–H Stretch. The peaks at 1641 cm^-1^ and 1637 cm^-1^ resemble N–H bend primary amines. The bands observed in 1163 cm^-1^ and 1170 cm^-1^ resemble C–N stretching alcohols. The FT-IR study revealed that the carbonyl group from proteins and amino acid residues has a strong ability to bend metal, suggesting that the proteins may be made of synthetic metal nanoparticles. As shown in Fig. FTIR spectrum A band at 1200–1800 cm^−1^ corresponded to the bending vibrations of the amide I and amide II bands of protein^40^.

The phytochemicals attached to the particle's surface may have served as capping agents to prevent agglomeration and stabilize the medium^41,42^. This implies that the creation and stabilization of te-AgNPs, gr-AgNPs, and am-AgNPs in the aqueous media may both be accomplished by biological agents. The solid assimilation groups at 3468.5 cm^−1^ and 3417.56 cm^−1^ compare to O–H extending vibration likewise, we additionally noticed solid pinnacles showing up at 1634.25 cm^−1^ and 1616.84 cm^−1^ in the IR spectra which are allocated as carbonyl stretch of amide-I bond and of –N–H stretch vibrations of amide-II bond demonstrating the presence of proteins. The trademark groups showed up at 2924.72 cm^−1^ and 2853.53 cm^−1^ compared to C–H for alkenes. Bands at 1384.53 cm^−1^ and 1354 cm^−1^ credited to C–H lopsided extending of alkanes. Bands at 1124.83 cm^−1^ and 1107.8 cm^−1^ are because of the C–OH extending of carboxylic corrosive. Additionally, tops saw at 780.76 cm^−1^ and 804.63 cm^−1^ were allotted to C–H extending of fragrant gathering, and the top at 521.22 cm^−1^ addresses the O–H bowing of phenol bunch.

The FTIR spectrum revealed the presence of carboxylic acid, and hydroxyl group dominantly along with C–N and N–H which are associated with primary as well as secondary metabolites. The extract usually contains enzymes and proteins like Superoxide dismutase (SOD), Catalase, Glutathione peroxidase (GPx), Glutathione reductase (GR) and Glutathione S Transferase^43^. In *Ocimum*, octa-aminopropyl may be present, potentially as a synthetic compound or a modification introduced to a protein. Its role could involve enhancing solubility, and stability, or facilitating interactions with other molecules^44^. Besides, –COOH and –OH functional groups are associated with flavanols, phenolics, and tannins. Previous literature has identified rosmarinic acid, chlorogenic acid, caffeic acid, aesculin, quercetin, luteolin, and apigenin from *Ocimum sanctum* that might contribute to carboxylic and OH groups^45^.

### Antimicrobial activity of synthesized AgNPs from *Ocimum* species

**Figure 5:**
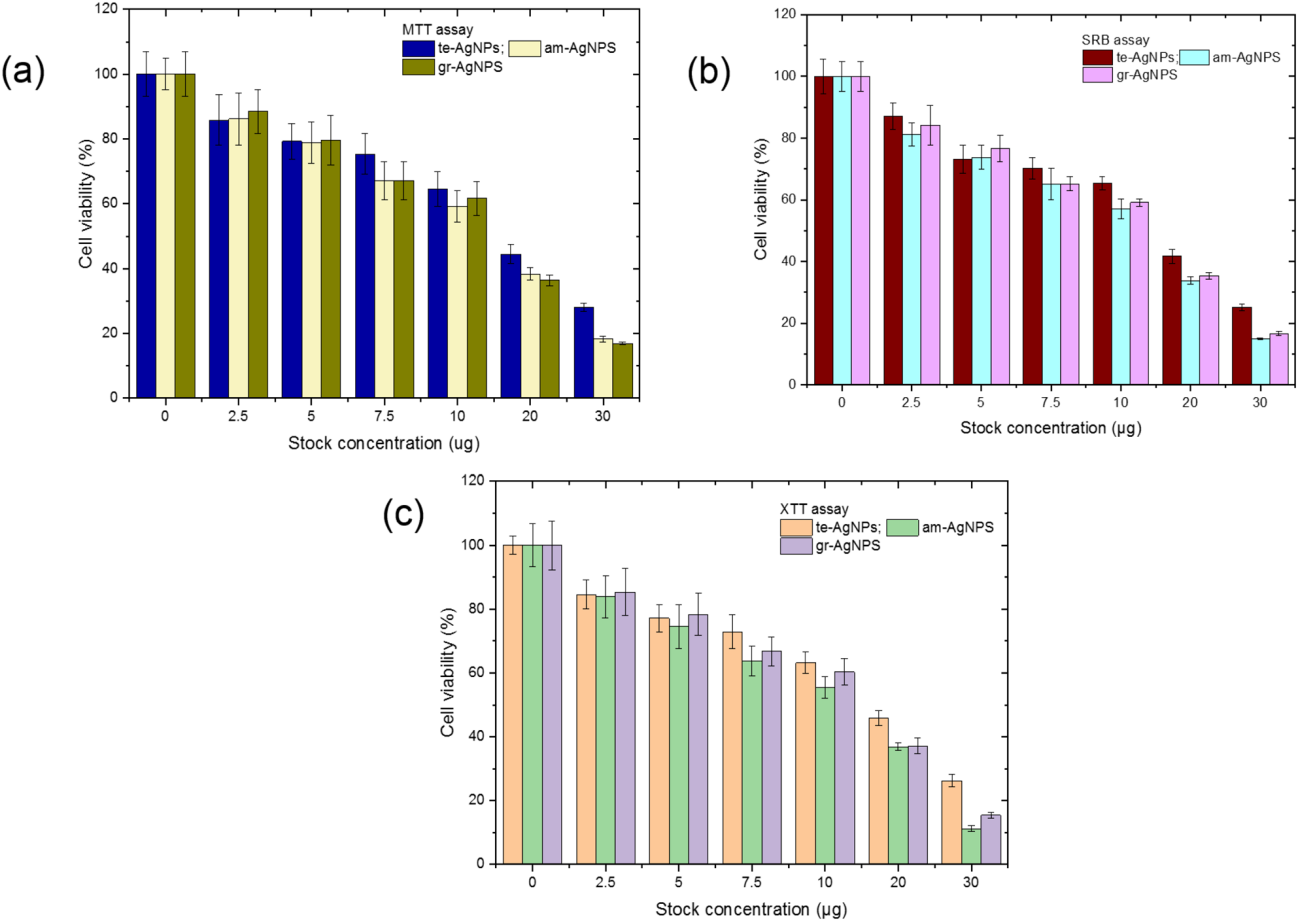
Cytotoxic effect of AgNPs from the *O. tenuiflorum*, *O. gratissimum*, and *O. americanum* leaf extracts on MCF-7 cells by (**a**). MTT assay; (**b**). XTT assay; and (**c**). SRB assay, (IC50 te-AgNPS: 18.04 ± 0.63 µg; gr-AgNPs: 15.41 ± 0.37 µg and am-AgNPs: 14.78 ± 0.89 µg). MTT: 3-[4,5-dimethylthiazol-2-yl]-2,5 diphenyl tetrazolium bromide; XTT: 2,3-Bis-(2-methoxy-4-nitro-5-sulfophenyl) -2H-tetrazolium-5-carboxanilide; SRB: Sulforhodamine B.

The antimicrobial assessments of the fabricated te-AgNPs, gr-AgNPs, and am-AgNPs showed the maximum inhibition zone (23 ± 1.58 mm) against *K. pneumoniae* whereas normal aqueous leaf extracts show (20 ± 1.15 mm) inhibition zone from am-AgNPs (Fig. 5). The *S. aureus* and *E. coli* showed 22 ± 1.80 mm and 20 ± 1.68 mm, respectively followed by (19 ± 1.50 mm) *B. subtilis* (Table 1). Antifungal properties of am-AgNPs synthesized from the *O. americanum* exhibited a maximum inhibition zone (26 ± 1.85 mm) against *A. niger* and 22 ± 1.40 mm against *C. albicans*. The te-AgNPs and gr-AgNPs of *O. tenuiflorum* and *O. gratissimum* respectively show the inhibition zones of 16 ± 1.2 mm against *S. aureus*. The gr-AgNPs showed 16 ± 1.12 mm against *S. aureus*, 14 ± 0.67 mm against *K. pneumoniae*, 15 ± 1.03 mm against *E. coli*, and 11 ± 0.54 mm against *B. subtilis*. Meanwhile, the antifungal results revealed 15 ± 0.80 mm against *A. niger* and 13 ± 1.05 mm against *C. albicans*. Data are demonstrated as mean ± SD of triplicates. The suitability of antimicrobial analysis, conducted with one-way ANOVA revealed the *F-value* and *Fcrit* of 60.04 and 2.25 respectively which suggested that the null hypothesis can be rejected and obtained results were statistically significant.

**Table 1:** Antimicrobial potential of respective AgNPs against selected microbial pathogens. Inhibition is measured as Inhibition Zone diameter in mm (mean ± SD). Amoxycillin and Ketoconazole was used as positive control for bacterial and fungal isolates respectively.

Microorganisms	*Ocimum tenuiflorum*		*Ocimum americanum*		*Ocimum gratissimum*		Negative control	Positive control
	AqE	te-AgNPs	AqE	am-AgNPs	AqE	gr-AgNPs		
	30 µg							30 µg
*E. Coli*	13 ± 1.13	14 ± 1.23	18 ± 1.42	20 ± 1.68	13 ± 1.00	15 ± 1.03	–	19 ± 1.50
*K. Pneumoniae*	10 ± 0.6	13 ± 1.06	20 ± 1.5	23 ± 1.58	12 ± 1.32	14 ± 0.67	–	18 ± 1.6
*S. aureus*	12 ± 0.85	15 ± 1.15	18 ± 0.98	22 ± 1.80	13 ± 0.51	16 ± 1.2	–	15 ± 1.05
*B. Subtilis*	10 ± 0.52	12 ± 0.47	14 ± 1.14	19 ± 1.50	8 ± 0.62	11 ± 0.54	–	13 ± 1.11
*A. niger*	10 ± 0.87	12 ± 0.33	21 ± 1.5	26 ± 1.85	8 ± 0.6	15 ± 0.80	–	13 ± 0.89
*C. albicans*	9 ± 0.41	11 ± 0.7	16 ± 1.4	22 ± 1.40	9 ± 0.45	13 ± 1.05	–	13.3 ± 0.9

### In vitro anticancer activity

**Figure 6:**
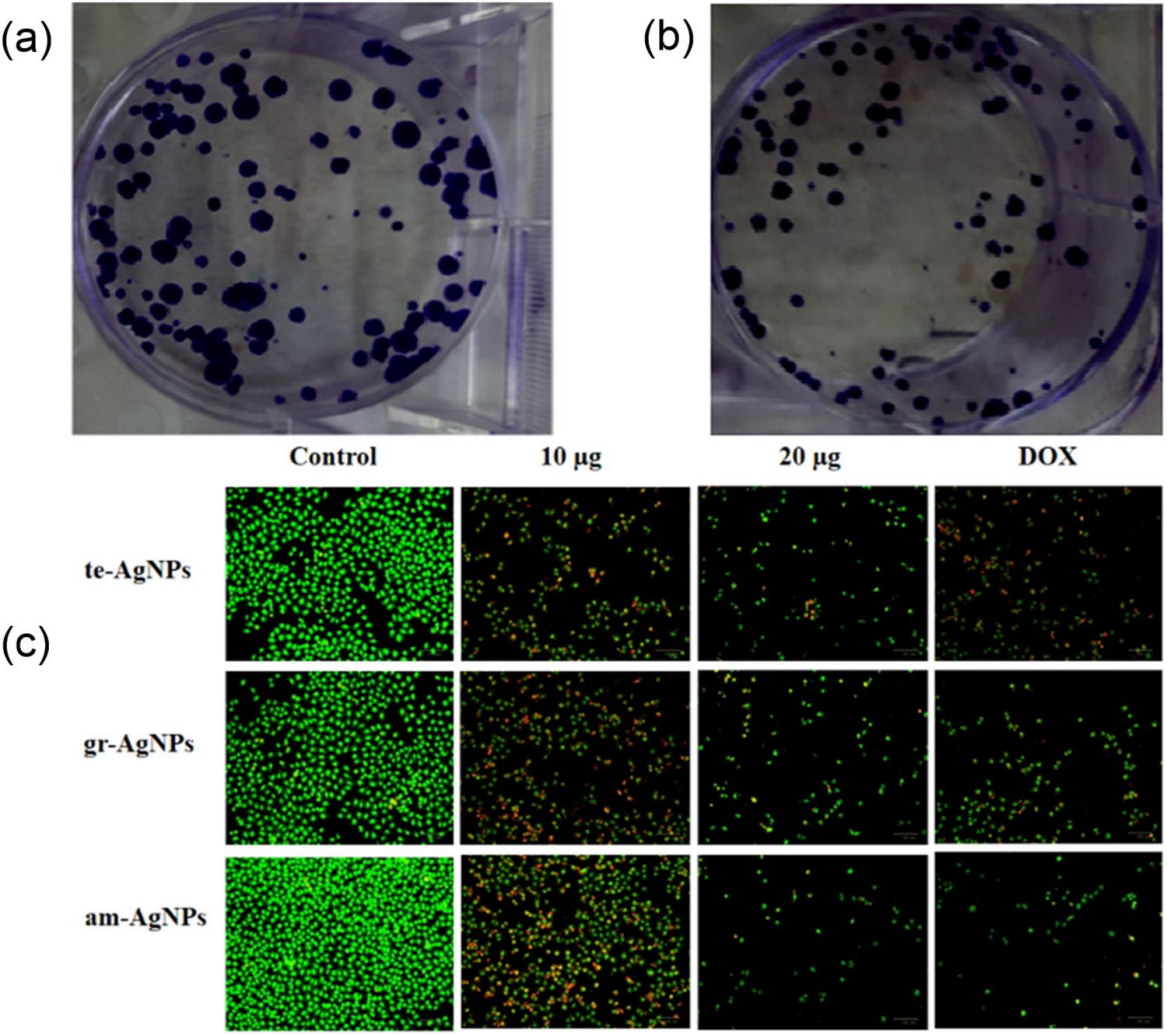
Effect of synthesized te-AgNPs, gr-AgNPs, and am-AgNPs (**a**) Clonogenic assay and (**b**) inducing apoptosis in the MCF-7 cells in MTT assay.

The influence of fabricated te-AgNPs, gr-AgNPs, and am-AgNPs from the *Ocimum* species has been investigated against the MCF-7 cells by four different methods including MTT assay, Sulforhodamine-B assay, XTT assay, and Clonogenic assay. Figure 5 shows the in-vitro cytotoxicity of synthesized te-AgNPs, gr-AgNPs, and am-AgNPs against MCF-7 cells via different methods. The results from the MTT assay indicate that aqueous extract-based nanoparticles (te-AgNPs, gr-AgNPs, and am-AgNPs) substantially diminished the viability of MCF-7 cells in dose dose-dependent manner. In comparison to te-AgNPs, gr-AgNPs, and am-AgNPs showed more pronounced cytotoxic activity and substantially inhibited the viability of MCF-7 cells (Fig. 5a). Similar patterns were also observed in other cytotoxic assays as well. The XTT assay (Fig. 5b), SRB assay (Fig. 5c), and Clonogenic assay (Fig. 6a–b) also showed dose-dependent cytotoxicity in MCF-7 cells. The IC_50_ value for te-AgNPs was 18.04 ± 0.63 µg, for gr-AgNPs, 15.41 ± 0.37 µg and for am-AgNPs, it was 14.78 ± 0.89 µg. Therefore, it was clear that am-AgNPs demonstrated more cytotoxicity in comparison to te-AgNPs as well as gr-AgNPs on MCF-7 cells. The apoptosis in cell demise is essentially portrayed by morphological changes like shrinkage of cells, chromatin build-up, core discontinuity, blebbing, and development of apoptotic bodies. Staining with AO and PI staining was used for the determination of live and dead cells. The control cells showed green fluorescence, whereas the synthesized te-AgNPs, gr-AgNPs, and am-AgNPs treated MCF-7 cells demonstrated an increased yellow and orange fluorescence, which indicates the occurrence of apoptotic cells. The DOX treatment also revealed increased apoptosis in the MCF-7 cells. The findings of AO/PI staining proved that the am-AgNPs treatment induced more apoptotic cell death in MCF-7 cells than the gr-AgNPs and te-AgNPs (Fig. 6c).

### Statistical analysis

Antimicrobial and anticancer analysis was assessed with ANOVA analysis using Microsoft Excel with the ‘Data analysis’ tool pack (Table 2). ANOVA analysis of the antimicrobial test revealed a P-value of 0.0016 (for rows) and 2E-20 (for table) which suggested the analysis was significantly suitable. A similar analysis was also conducted against MCF-7 cell lines via MTT, XTT, and SRB assay. ANOVA analysis was conducted with individual tests. The P-values for rows and columns were 6.5E-10 and 0.025 for MTT assay, 1.07E-09 and 0.02 for XTT assay, and 3.07E-10, 0.02 for SRB assay. In addition, the IC_50_ value was calculated for individual tests followed by the average IC_50_ calculation. Average IC_50_ values were 18.04 ± 0.63 (for te-AgNPs), 14.77 ± 0.89 (am-AgNPs), and 15.42 ± 0.37 (gr-AgNPs). To find the best AgNPs against cancer cells were determined by two-way ANOVA analysis between three groups. The *P* values for the IC_50_ were 0.037 and 0.000771 which not only identified the significance of the model but also suggested that am-AgNPs have significantly higher anticancer potential among the group.

**Table 2:** Statistical analysis of anticancer activity of te-AgNPs, gr-AgNPs and am-AgNPs by Analysis of Variance (ANOVA).

Source of variation	Df	Sum of squares	Mean squares	F Values	*P* value	Fcrit
Antimicrobial analysis						
Rows	5	68.50938	68.50938	4.913635	0.001646	2.485143
Columns	7	1745.305	1745.305	89.41206	2E − 20	2.285235
Anticancer analysis						
MTT assay						
Rows	5	9334.055	1866.811	222.5921	6.5E − 10	3.325835
Columns	2	91.53243	45.76622	5.457006	0.024993	4.102821
XTT assay						
Rows	5	6469.263	1293.853	201.3545	1.07E − 09	3.325835
Columns	2	70.94941	35.47471	5.520714	0.024245	4.102821
SRB assay						
Rows	56932.403	6932.403	1386.481	258.9191	3.07E − 10	3.325835
Columns	260.96168	60.96168	30.48084	5.692162	0.022363	4.102821
IC_50_						
Sample	te-AgNPS		am-AgNPs		gr-AGNPs	
IC_50_	18.04 ± 0.63		14.77 ± 0.89		15.42 ± 0.37	
Rows	2	2.145225	1.072613	8.382651	0.037106	6.944272
Columns	2	17.92585	8.962922	70.04676	0.000771	6.944272

### Chemical profiling of extract by GC–MS

**Figure 7:**
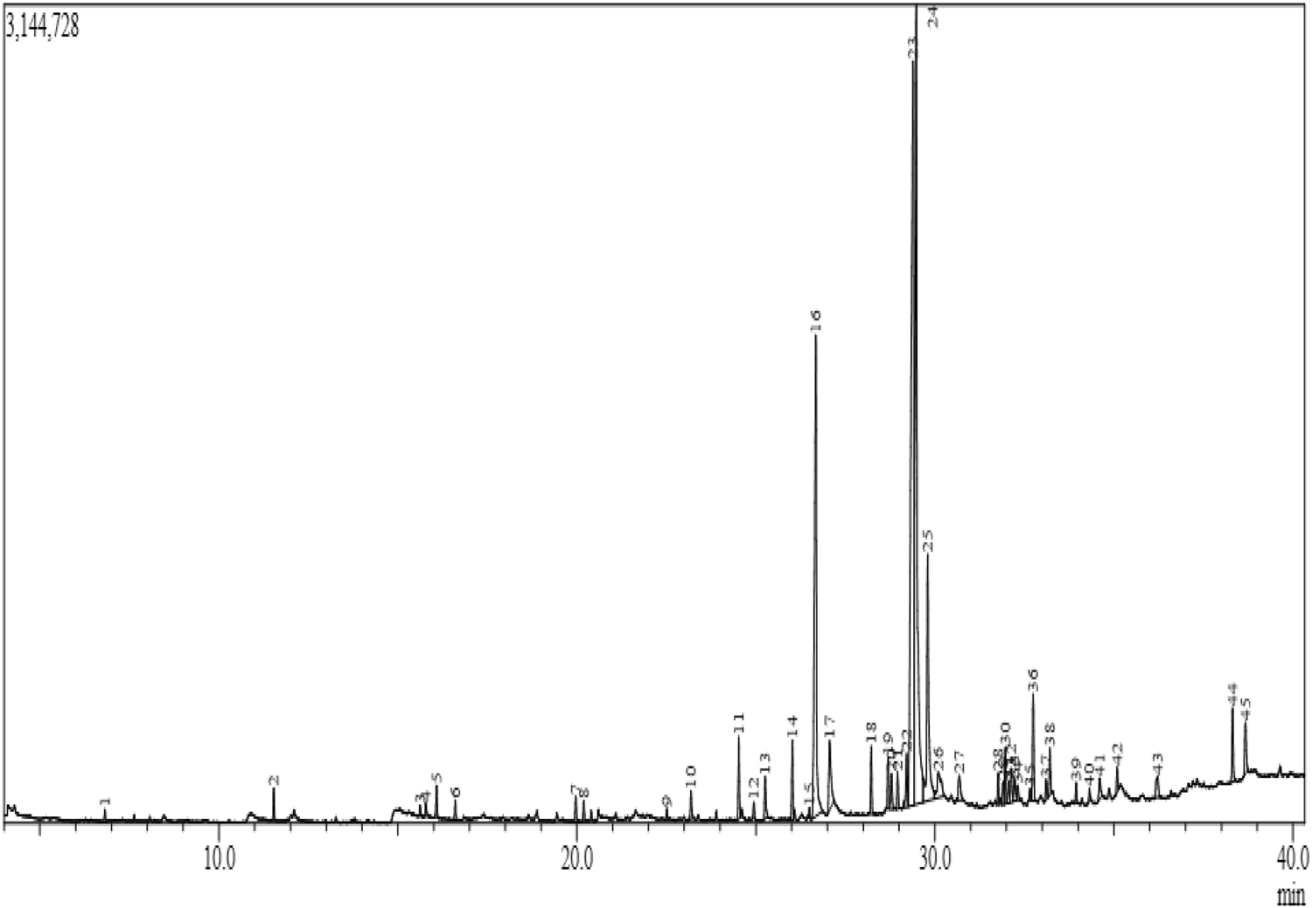
Gas chromatogram of *Ocimum americanum.*

*Ocimum americanum* has shown the maximum efficiency in cancer inhibition among all three extract-based nanoparticles hence chemical profiling was done via GC–MS for the identification of compounds. The scanning was done for upto 40–33 min and with the molecular weight range of 50–500 da (Fig. 7). Total 45 peaks were identified from the extract out of which only 12 peaks have area of more than 1%. Octadecadienoic acid (7.05) and its derivative (27.99%), oleic acid (23.82%), and hexadecenoic acid (13.53%) have occupied more than 50% area on the peaks (Table 3). These compounds are responsible for the bioactivity of the extract against microorganisms as well as cancer cells. 9,12-Octadecadienoic acid derivatives are one of the most prominent oil/polyunsaturated fatty acids in plants and have bioactive potential^46,47^. Oleic acid is a monounsaturated fatty acid that has a direct role in cancer management. It has shown anti-proliferative activity that aids in cancer inhibition. Besides, it also suppresses inflammation by modulating the production of inflammatory mediators and VEGF effector pathway^48^. Abietic, palustric, and eicosanoic acids were also detected in extract and have also been reported for their applications in pharmaceuticals.

**Table 3:** GCMS profile of *Ocimum americanum* (the retention time and area occupied in chromatogram).

Peak	Retention time	Area%	Compound name
1	6.814	0.17	Decane
2	11.533	0.46	Dodecane
3	15.619	0.17	3,5-Diisopropoxy-1,1,1,7,7,7-hexamethyl-3,5-bis(trimethylsiloxy)tetrasiloxane
4	15.78	0.24	2-Propenoic acid, 3-Phenyl-, methyl ester
5	16.081	0.5	Tetradecane
6	16.604	0.29	Bicyclo[7.2.0]undec-4-ene, 4,11,11-trimethyl-8-methylene-, [1R-(1R*,4E,9S*)]
7	19.968	0.4	(-)-5-Oxatricyclo[8.2.0.0(4,6)]Dodecane,12-TriMethyl-9-Methylene-, [1R-(1R*,4R*,6R*,10S*)]-
8	20.192	0.31	Tetradecane
9	22.516	0.17	Octadecanoic acid, Methyl ester
10	23.187	0.53	Tetradecanoic acid
11	24.522	1.29	Neophytadiene
12	24.942	0.27	Neophytadiene
13	25.257	0.84	3,7,11,15-Tetramethylhexadec-2-en-1-yl acetate
14	26.019	1.26	Hexadecanoic acid, methyl ester
15	26.496	0.14	Dibutyl phthalate
16	26.674	13.53	n-Hexadecanoic acid
17	27.06	2.16	1H-inden-1-one, 2-(2-furanylmethylene)-2,3-dihydro-
18	28.223	1.13	7-Isopropyl-1,1,4a-trimethyl-1,2,3,4,4a,9,10,10a-octahydrophenanthrene
19	28.691	0.81	9,12-Octadecadienoic acid (Z,Z)-, methyl ester
20	28.788	0.73	9,12-Octadecadienoic acid (Z,Z)-, Methyl ester
21	28.958	0.62	2-Hexadecen-1-ol, 3,7,11,15-Tetramethyl-, [R-[R*,R*-(E)]]-
22	29.207	0.96	Octadecanoic acid, Methyl ester
23	29.39	27.99	9,12-Octadecadienoic acid (Z,Z)-
24	29.479	23.82	Oleic Acid
25	29.798	7.05	Octadecanoic acid
26	30.095	1.49	9,12-Octadecadienoic acid (Z,Z)-
27	30.682	0.76	9,12-Octadecadienoic acid (Z,Z)-
28	31.765	0.47	2-Propenoic acid, 3-phenyl-, methyl ester
29	31.899	0.44	(E)-Zimtsaeure, Methyl ester
30	31.965	0.96	2-Propenoic acid, 3-phenyl-, methyl ester
31	32.056	0.42	Andrographolide
32	32.133	0.67	1-Phenanthrenemethanol, 1,2,3,4,4a,5,6,9,10,10a-decahydro-1,4a-dimethyl-7-(1-methylethyl)-, [1R-(1.alpha.,4a.beta.,10a.alpha.)]
33	32.229	0.57	9,12-Octadecadienoic acid (Z,Z)-
34	32.312	0.28	(2E,4E,12E)-13-(Benzo[d][1,3]dioxol-5-yl)-N-isobutyltrideca-2,4,12-trienamide
35	32.651	0.17	Eicosanoic acid
36	32.744	1.96	1-Phenanthrenemethanol, 1,2,3,4,4a,9,10,10a-octahydro-1,4a-dimethyl-7-(1-methylethyl)-, [1R-(1.alpha.,4a.beta.,10a.alpha.)]-
37	33.102	0.42	Abietic acid
38	33.21	0.85	Palustric acid
39	33.946	0.33	((1R,4aR,4bR,10aR)-7-Isopropyl-1,4a-dimethyl-1,2,3,4,4a,4b,5,6,10,10a-decahydrophenanthren-1-yl)methanol
40	34.315	0.21	Pregnan-17,21-diol-20-one, 3,9-epoxy-3-O-methyl-11-thiocyano-,
41	34.603	0.54	Hexadecanoic acid, 2-hydroxy-1-(hydroxymethyl)ethyl ester
42	35.09	0.46	(1S,3aR,4R,8R,8aS)-1-Isopropyl-3a-methyl-7-methylenedecahydro-4,8-epoxyazulene
43	36.221	0.7	5.alpha.-Pregn-16-en-20-one, 12.beta.-hydroxy-, acetate
44	38.315	1.31	Squalene
45	38.675	1.16	2-Butene-1,4-dione, 1,2,3,4-tetraphenyl-, (Z)-

## Discussion

In contrast to bulk materials, NPs have distinct physical, chemical, and biological characteristics, including crystal structures, and specific targeting and controlled release features. Drug delivery, aesthetics, tissue engineering, theranostics, and agriculture are just a few of the industries that are fast-growing NP applications^49^. AgNPs are gaining more and more attention for their numerous uses as antimicrobial, antioxidant, and anticancer, as well as for improving vaccine immunogenicity^50^. Nanoparticles, particularly AgNPs have been widely utilized as antimicrobial sources in the medical field. AgNPs are more effective against microbes than bulk silver due to their colossal specific surface area to surface atom ratio^51^. As a result of their biological activities, AgNPs are employed in the battle against multidrug-resistant bacteria. Researchers looked at the fabrication of AgNPs for effective cancer therapy, inquiry, and diagnostics^52^. To increase AgNPs' sensitivity and effectiveness against different tumor cells, they can be coupled to or loaded with different drugs, polysaccharides, and nanostructures^53^. The development of new antimicrobial drugs has been encouraged by the emergence of multidrug-resistant pathogens. AgNPs have powerful antimicrobial properties against several pathogens.

In addition, AgNPs have substantial anticancer properties against several cancers including lung, hepatocellular, breast, and colorectal in both in vitro and in vivo settings^54^. Due to their capacity for oxidation and reduction, biomolecules like polyphenol and its derivatives were used to reduce Ag^+^ ions into AgNPs^55^. It was highlighted that plant metabolites have exceptional efficiency in the bio-reduction of Ag^+^ ions and the stability of AgNPs^56^. AgNPs from plants contain phytochemical substances that may reduce silver ions; however, this process is currently unknown. In this study, we witnessed that the te-AgNPs, gr-AgNPs, and am-AgNPs fabricated from the aqueous leaf extracts of *Ocimum* leaf extracts were found effective against the clinical pathogens and breast cancer MCF-7 cells.

AgNPs generally have an antimicrobial effect, although the exact mechanism by which they do so is unknown. However, it has been proposed that the mechanism by which they are antibacterial is the binding of negative charge on the bacterial cell membrane to positive charge Ag^+^ ions, which may cause the bacterial cell to die. According to this, AgNPs can cause damage to membranes, which can either impede bacterial growth or cause bacteria to die^57^. Similar to this, AgNPs can saturate and cling to fungal hyphae and generate insoluble compounds that bind to lipids and enzymes, rupturing the membrane and resulting in cell lysis^58^.

AgNPs have been shown to have antibacterial action through a number of methods, including direct bacterial cell membrane destruction. Increased membrane permeability and DNA damage are brought on by the discharge of Ag^+^ ions, which also generate reactive oxygen species^59^. AgNPs' cytotoxicity and antibacterial activity, as well as their capacity for penetrating cells, are all greatly predisposed by their size and surface chemistry, as has been demonstrated. As a result, choosing the right stabilizing reducing agents can increase the nanoparticles' antibacterial activity, which can therefore result in a reduction in the therapeutic dose^60^. Additionally, the functionalization of AgNPs with natural organic compounds known to have antimicrobial activity may enhance such effects, which are referred to as capping agents. By preventing AgNPs aggregation, these capping agents may also enhance the AgNPs' colloidal stability and have a substantial impact on how they interact with other in vivo components^61^.

Our results indicated that Gram-negative bacteria were more vulnerable than Gram-positive species^62^. This is because of the thin peptidoglycan layer and additional lipopolysaccharide outer membrane present in the Gram-negative strain, which suggests the existence of a periplasmic membrane layer. The NPs entry and discharged ions into the cell might be facilitated by this arrangement. The thick coating of peptidoglycan seen in the cell membranes of Gram-positive bacteria, however, contains covalently bonded teichoic and teichuronic acids and may serve as a shield against the AgNPs^63^. In this study, our findings demonstrated that the fabricated te-AgNPs, gr-AgNPs, and am-AgNPs efficiently decreased the growth of different pathogenic bacteria and fungi, which is witnessed by the higher inhibitory zones around the AgNPs-loaded wells. The am-AgNPs treatment more effectively inhibited the growth of the pathogens than the te-AgNPs and gr-AgNPs. The subsequent production of extracellular ROS disrupts the cell's natural antioxidant defense, leading to a higher cell wall injury or cell necrosis via impairing ATP synthesis and DNA replication^64^. Two potential mechanisms of toxicity include inducing the production of ROS, which results in DNA injury and apoptosis. The Ag^+^ ions in the cytosol interfere with natural metabolic and cell cycle systems, they are primarily responsible for cell death^65^.

Additionally, several studies have shown that the NPs' surface morphology significantly affects how active they are; NPs' small size and high surface area facilitate cell penetration. It appears that NPs with a small diameter and a high dosage of Ag^+^ ions can enter the intracellular space of bacteria^66^. It was highlighted that cells administered with AgNPs can produce higher ROS than cells administered with Ag^+^ ions alone, leading to the development of oxidative stress. Ag^+^, however, inhibits the site and raises ROS, which can damage DNA without obviously disrupting the membrane, signifying a complex toxicity strategy for inhibiting the growth of tumor cells^67^. *Ocimum* species exhibited cytotoxic activity on the MCF-7 cell line^68^. Cell death expanded with expanding groupings of AgNPs and hindrance of half-cell passing requires a little portion of AgNPs. It has been accounted for that the AgNPs treatment causes harm to the mitochondrial film and delivers cytochrome c, which is a basic angle showing apoptosis in cells animated by cytoplasm^69^. Here, our outcomes demonstrated that the formulated te-AgNPs, gr-AgNPs, and am-AgNPs effectively inhibited growth and induced apoptosis in the breast cancer MCF-7 cells. Apoptosis and growth retardation incidence were observed in am-AgNPs treated MCF-7 cells in all cytotoxicity tests revealing the potent invitro anticancer activity of am-AgNPs higher than the te-AgNPs and gr-AgNPs. Hence, it was clear that the am-AgNPs from the *O. americanum* species are effective in inhibiting the pathogenic bacterial and fungal growth and induction of apoptotic cell death in the MCF-7 cells.

## Materials and methods

### Collection of plant materials

The fresh plant materials of *O. tenuiflorum, O. gratissimum*, and *O. americanum* were collected from the Kulumani (Latitude: 10.80915; Longitude: 78.59011), Kallikudi (Latitude: 10.9772; Longitude: 78.9515), and Palkalaiperur villages of Tiruchirappalli district in the month of April to May. During the collection period, the temperature, and humidity of 36 ± 5 °C and 60 ± 5% respectively. The collected plant materials were taxonomically identified by Dr. S. Soosairaj, Department of Botany, St. Joseph’s College (Autonomous) Tiruchirappalli. National guidelines and legislation were strictly followed for the sample collection of plant sample. The voucher specimens of the collected plant materials were deposited at the Rapinet Herbarium Centre (Herbarium Accession numbers—2794, 2793, and 2792, respectively).

### Preparation of *Ocimum* leaf extract

To make leaf extract, the leaves were gathered in a beaker and twice rinsed with deionized water to discard the dust that remained. 100 ml of double-distilled water and 10 g of properly cleaned dried leaves were crushed together in a mortar and pestle^70^. The aqueous extract was pulverized before being added to a 250 ml beaker and heated there for 10 min at 80 °C. After cooling to 37 °C, the extract was filtered and utilized to synthesize AgNPs.

### Green synthesis of AgNPs

The AgNPs synthesis is a one-step procedure. The mixture was heated at 80 °C for 15 min after being blended with 90 ml of AgNO_3_ and 10 ml of leaf extracts. The solution color reformed from pale yellow to dark brown due to the development of AgNPs. The reaction mixture was centrifuged at 15,000 rpm for 20 min at 4 °C to separate the generated AgNPs (te-AgNPs for *O. tenuiflorum*, gr-AgNPs for *O. gratissimum*, and am-AgNPs for *O. americanum*). The AgNPs pellet was collected and washed with deionized water to remove phytochemical and silver ions residues and then AgNPs were lyophilized and stored in a cool, and dry environment till further use^71^.

### Characterization of synthesized AgNPs

To recognize the development of AgNPs in the suspension, the UV–visible spectroscopic analysis was performed using Perkin Elmer equipment. A wavelength between 200 and 1000 nm was utilized to investigate the sample. The size and shape of AgNPs prepared from AgNO_3_ using *Ocimum* extract were confirmed by scanning through a Scanning electron Microscope (SEM; Carl Zeiss Ultra 55 FESEM). Besides, the particle size and distribution were confirmed by the DLS-Zeta sizer (Micromeritics).

The AgNPs were prepared with plant extract hence nanoparticles ay accompanied by biomolecules. The presence of biomolecules was assessed by FT-IR (Perkin Elmer) analysis by which available functional groups can be identified. For analysis, the sample powder was mixed with the KBr disc technique, and the spectrum was recorded at 4000–500 cm^−1^.

### Antimicrobial assay

The clinical pathogens were acquired and collected from the Department of Microbiology, Bharathidasan University, Tiruchirappalli. The synthesized AgNPs were dissolved in sterile DMSO and used as a stock (1 mg/mL) for antimicrobial testing. The antimicrobial potential of AgNPs was evaluated by the well diffusion technique^71^. Different concentrations of AgNPs (40, 50, and 60 µg/disk) were prepared with dried AgNPs in Milli Q water (1 mg/mL) and applied to the microbiological strains of *S. pneumoniae, E. coli, K. pneumoniae, S. aureus, A. niger*, and *C. albicans*. Amoxicillin and ketoconazole (1 mg/mL) were used as a positive control against bacteria and fungi respectively. The inhibition zones were recorded following a 24 h treatment period.

### In vitro anticancer assays

#### MTT(3-[4,5-dimethylthiazol-2-yl]-2,5 diphenyl tetrazolium bromide) assay

The DMEM enhanced with 10% FBS was utilized to maintain the MCF-7 cells, which were then kept at 37 °C in an incubator with 5% CO_2_. The growing cells were trypsinized and used for further experiments after it had reached 80% confluency. Cytotoxicity analysis of AgNPs was conducted using MTT assay^72^ by confirming the viability of MCF-7 cells followed by AgNPs administration. In order to test their ability to adhere, the cells were loaded at a population of 5 × 10^3^ cells per well in a 96-well plate that had been filled with DMEM. The growth medium of the cells was then refilled and administered with different dosages of AgNPs (2.5, 5, 7.5, 10, 20, and 30 μg) for 24 h at 37 °C. Following the treatment phase, 20 μl of MTT and 100 μl of DMEM were mixed in each well and kept there for 4 h at 37 °C. To liquefy the generated formazan stone, 100 μl of DMSO was later mixed into the wells. By determining the absorbance at 570 nm, the cell viability was assessed. The apoptosis in MCF-7 cells after treatment with AgNPs was determined by acridine orange/ethidium bromide (AO/EB) staining^73^. In brief, 5 × 10^3^ MCF-7 cells were loaded into each well of a 24-well plate, and the cells were then maintained for 24 h at 37 °C. Following that, cells were supplemented with AgNPs at doses of 10 and 20 μg and 2 μg of doxorubicin (DOX) as a standard drug, and they were then maintained for 24 h at 37 °C. The cells were then stained for 5 min using 100 μg/ml of AO/EB stain in a 1:1 ratio after the treatment period. Using a fluorescent microscope, the apoptosis in treated cells was evaluated.

#### (2,3-Bis-(2-methoxy-4-nitro-5-sulfophenyl)–2H-tetrazolium-5-carboxanilide (XTT assay)

XTT assay relies on the activity of mitochondrial dehydrogenase enzyme from the viable cells. The active enzyme can act on the tetrazolium ring and soluble formazan will be formed as product^74^. In comparison to other tests, the XTT assay is highly sensitive and accurate however it uses a highly toxic XTT reagent^75^. In order to test the viability, 5 × 10^3^ cells per well were loaded in a 96-well plate with DMEM. Different dosages of AgNPs (2.5, 5, 7.5, 10, 20, and 30 μg) were added to separate wells and incubated at 24 h at 37 °C. Post-incubation, 100 µL of prepared XTT reagent was added and incubated at 37 °C for 4 h. Absorbance was recorded at 450 nm and activity was assessed.

#### SRB (Sulforhodamine B) assay

Sulforhodamine B assay is one of the most reliable methods to assess drug-induced cytotoxicity with high accuracy and affordability. SRB is an amino xanthene dye with two sulfonic groups that can bind with amino residues of cellular proteins^76,77^. The estimation can be done by following the method described by Vichai and Kritikara^76^. MCF-7 cells (5 × 10^3^ cells) were added to each well of 96-well plate and fixed with 25 μL of 10% (wt/vol) TCA solution and incubated for up to an hour at 4 °C. After fixing, cells were washed slowly and dried with paper towels. 100 µL of SRB dye solution (0.057% wt/vol) was added and mixed to each well and incubated for 30 min. Unbounded dye was removed by washing with 1% (V/V) acetic acid. The protein-bound dye was dissolved in 200 µL of a 10 mM Tris base solution (pH 10.5) and quantified at 510 nm.

#### Clonogenic assay

The Clonogenic assay relies on the fact that each cell can grow into a larger colony by division. The assay was earlier designed to test the efficiency of radiation on cancer cells but later it was adopted for evaluating the cytotoxic effect of drugs on cancer cell^78–80^. The assay was performed by following the protocol of Munshi et al.^78^. MCF-7 cells (5 × 10^3^ cells per well) were added to each well as well plate with complete media and incubated at 37 °C for 24 h. The cells were treated with different dosages of AgNPs (2.5, 5, 7.5, 10, 20, and 30 μg) for 24 h at 37 °C. The medium was replaced and incubated for a week to attain the ideal colony size and density. Cells were gently washed with 1X PBS and fixed with 500 µL of 100% methanol at − 20 °C for 15–20 min. Remove methanol by aspiration and wash the cells with 1X PBS 3–4 times. The cells were stained with 500 μL of Coomassie blue and incubated for 15–30 min. Unbounded stain was removed with 1X PBS and colonies were counted.

### Statistical analysis

The fitness of the analysis related to antimicrobial and anticancer activity was determined by two Analysis of Variance (ANOVA) analyses. The analysis was conducted with Microsoft Excel using a data analysis tool pack. The model fitness was determined with a p-value which must be < 0.05 to consider a model significant.

### Chemical profiling of extract with GC–MS

The phytochemical constituents in *Ocimum* leaf extract were analyzed and identified using GC–MS (Perkin Elmer Clarus 500, Shelton, CT, USA) following the method described^81^. For analysis, Helium gas was used as mobile/carrier and operated at a flow rate of 1.20 mL/min. The temperature of the injection port and oven were kept at 250 °C and 280 °C. The mass spectrum analysis was conducted with ion source and interface temperature of 200 °C and 250 °C respectively. The spectrum was recorded between 4–40.33 min with full scan mode considering the range of 50–500 Daltons. The compound peaks were identified with the standard libraries from NIST (The National Institute of Standards and Technology) and Wiley compound libraries.

## Conclusion

The synthesized te-AgNPs, gr-AgNPs, and am-AgNPs from the *O. tenuiflorum, O. gratissimum*, and *O. americanum*, respectively by the green route method effectively decreased the growth of the pathogenic strains. Furthermore, the synthesized te-AgNPs, gr-AgNPs, and am-AgNPs substantially inhibited cell growth and triggered apoptotic cell death in the MCF-7 cells. The am-AgNPs treatment revealed more antimicrobial and potent in vitro anticancer activity on MCF-7 cells than the te-AgNPs and gr-AgNPs treatments. As far as is known, the plant material employed in the green synthesis of nanoparticles was first used. It is a common weed. It is noteworthy that *Ocimum americanum* can produce finer AgNPs than the other species in this genus. The approach was original and economical. To support its additional therapeutic use, we strongly recommend additional studies in the future.

## Data Availability

The data used to support the findings of this study are included within the article. Further information can be made available upon request from corresponding authors.
